# Impact of SARS-Con-V2 on the Poultry Industry in Kuwait: A Case Study

**DOI:** 10.3389/fvets.2020.577178

**Published:** 2020-09-22

**Authors:** Hanan Al-Khalaifah, Afaf Al-Nasser, Noura Abdulmalek, Hamad Al-Mansour, Abdulaziz Ahmed, Gehan Ragheb

**Affiliations:** Environment and Life Sciences Research Center, Kuwait Institute for Scientific Research, Kuwait City, Kuwait

**Keywords:** crisis, curfew, Kuwait, poultry industry, SARS-Con-V2

## Abstract

The SARS-Con-V2 crisis influenced all aspects of life in the country including academics, economics, medical, and other fields. Poultry industry, whether locally or globally, is one of the investment fields that has been affected by the current SARS-Con-V2 crisis. As part of this global world, the local poultry companies in Kuwait have been impacted by restricting movements and transportation of the employees in between the company branches, exportation and importation of goods, bank closures, rise in the imported and local vaccination and drug prices, sales and marketing. The current case study paper sheds light on the impact of the current SARS-Con-V2 crisis on the local poultry industry, as well as the challenges and gaps in the local poultry value chain. It also propose solutions to mitigate this impact and to enhance sustainability of poultry products during other future global or local crisis.

## Introduction

The continuous increase in the global human population increases the demand for poultry meat and eggs. These are the most widespread healthy food items worldwide because of many reasons such as being major sources for high quality protein, essential vitamins, minerals, and amino acids, as well as being relatively cheap when compared to other food sources. Poultry production has been identified as a major element that contributed to food security worldwide (1–3).

The World Food Summit in 1996 has defined food security as “a situation when all people, at all times, have physical and economic access to sufficient, safe, and nutritious food to meet their dietary needs and food preferences for a healthy and active life” (4). This healthy supply of poultry meat and eggs normally occurred through a poultry value chain that links all the poultry production elements to the final consumer. The elements of a poultry value chain involve planning, production, transport, processing, packaging, storing, retailing, quality control, and final transport to the consumer. An industry value chain is a physical representation of the various processes involved in producing goods (and services), starting with raw materials and ending with the delivered product. The value chain is a tool used to identify insufficiencies in the Kuwait poultry industry and opportunities for problem solving and possible creation of small to medium enterprises. It is essential to understand the detailed features of each of these elements to ensure smooth poultry value chain. The coronavirus has a great impact on all aspects of the daily life, including all the elements of the poultry value chain of both eggs and broiler meat (5, 6).

### Retrospective Data and Information of Poultry Production in Kuwait Before COVID 19 Crisis

Poultry industry is one of the most important agricultural sectors in Kuwait. The local consumption of poultry meat and eggs is quite high compared to other countries. The average consumption of chicken meat in Kuwait (2004–2016) was 64.4 Kg/cap/year. The average egg consumption is 14.94 kg/cap/year. Most of the Kuwait consumers prefer the locally produced meat (live and chilled) because of freshness and its compliance to the Islamic Halal slaughter way. However, the local meat is being sold at about 50% higher prices than the imported one, which is acceptable by the local consumers. About 60% of the local broiler meat is sold as live, 5% as chilled and the rest frozen (7–10).

Poultry producers export eggs to the other gulf countries and Iraq as the local self-sufficiency of table eggs is 101%. On the other hand, Kuwait import about 80% of the local consumed broiler meat as the local self-sufficiency is only 20% (9). Kuwait nationals (only 40% of the population) prefer poultry produced locally to that imported.

Feed constitutes a major cost for poultry producers in Kuwait. Most of the feed ration ingredients are imported from other countries. This represents a challenge for the local producers in terms of fluctuating prices and quality. However, the governmental Kuwait Flour Mills Company sells subsidized corn to the producers. The conversion rate in the local farms ranges from 2 to 1. The slaughter age of the chickens in Kuwait is usually 35 days (9, 11).

The State of Kuwait is a small country situated in the northern edge of Kingdom of Saudi Arabia at the tip of the Arabian Gulf. The COVID 19 pandemic has been officially reported in Kuwait on February 24, 2020 in a patient came from Iran. As of June 13, 2020, there were 35,466 confirmed cases of SARS-Con-V2 with 289 deaths. Since March 10, 2020, Kuwait started its subsequent partial and full curfews for its population of 4.5 million people. The partial curfew started March 10, 2020 until April 20, 2020 as a 11-h ban from 5 p.m. to 4 a.m. Kuwait then expanded the partial curfew to be a for 16 h a day, from 4 p.m to 8 a.m. from April 21, 2020 until May 9, 2020. Then, the full curfew was applied for 20 days on May 10 to May 30, 2020. The partial curfew then returned from May 31, 2020 until writing this paper on June 26, 2020.

These curfews, whether partial or full, affected all aspects of life in the country including academics, economics, medical etc. As in any other country worldwide, the local poultry industry is one of the agricultural sectors that has been strongly affected by the suspension and the lock down occurred as a results of the SARS-Con-V2 crisis (12).

The objective of the current paper is to shed light on some aspects that have been affected in the poultry companies so far as a result of the current COVID 19 crisis. This objective will be approached through surveying, collection, and analysis of information from the major poultry companies in Kuwait that supply the local society with their needs of table-eggs and poultry meat. Some retrospective data of the poultry industry in Kuwait before the COVID 19 crisis are also presented.

## Methodology

The objective of the current research study is to investigate the impact of COVID 19 on the poultry industry in Kuwait. Retrospective data were first collected to retrieve some information related to the status of the local poultry industry before and after the COVID 19 crisis. The methodology and design of the study also involved identification of the major poultry companies in Kuwait and contacts with the owners and farm supervisors for data collection. The Public Authority for Agricultural Affairs and Fisheries (PAAFR) was contacted to collect the latest information related to the existing poultry companies in Kuwait. PAAFR is an authorized governmental organization in Kuwait that administers the local poultry companies. It is also responsible of offering subsidies for feed and provides vaccination programs for the major local poultry diseases. Farm visits were avoided for security reasons related to the quarantine restriction by the Kuwait government.

A data analysis sheet was prepared and was electronically sent to the company owners and operation/production managers to fill in the information related to their companies during the COVID 19 crisis. In addition, intensive contacts were done by the phone to confirm and elaborate the results. The testing period was 16 days between May 25 and June 30, 2020.

### Major Poultry Farms in Kuwait

**PLATE 1:**
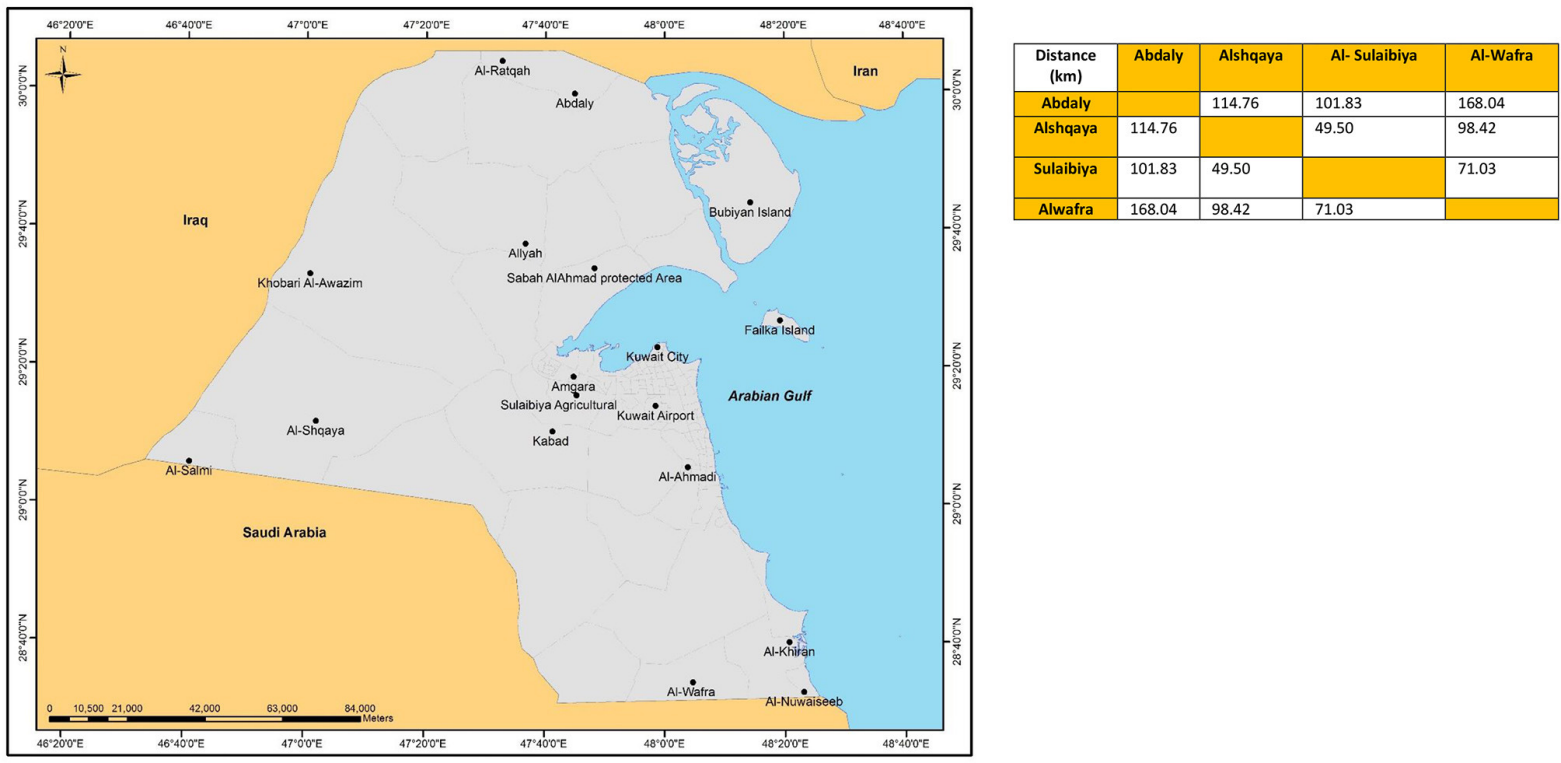
Locations of the Poultry Farms in Kuwait.

The major poultry farms in Kuwait are specialized farms for production of poultry meat, table eggs, and hatching eggs. These are located in different locations in Kuwait to ensure enough distances between them for biosecurity purposes. These locations are Al-Wafra, Al-Sulaiybia, Al-Abdali, and Al-Shagaiya. Plate 1 shows the distribution of these locations on Kuwait's map. There are about 20 specialized farms in these areas. These farms belong to eight local poultry companies. The names of these farms were kept anonymous for the sake of confidentiality requested by the farm owners. The farms were assigned numbers from 1 to 8. Companies from 1 to 3 are considered large companies that provide the Kuwait society with most of its needs of table-eggs and broiler chickens. Companies from 4 to 6 are medium companies and those numbered 7 and 8 are small companies.

The criteria used for the farm size as small, medium, and large is based on the production capacity of the farm. Small farms include few houses (1–2 houses) with production capacity from 5,000 to 10,000 birds in one cycle in one house (30,000 to 60,000 birds in 1 year/house), so maximum production capacity of small farms is 60,000 to 120,000 birds per year. Medium-sized farms contain more houses with more bird capacity than the small farms, and large farms are the integrated companies that produce most of the local production from meat and eggs. Table 1 shows that the seven large-sized specialized poultry farms exist with production capacity of more than 1 million broilers per year, eight medium size farms with production capacity of <1 million per year and six small-sized farm of 120,000 birds or less. Data in the table indicates that only one farm is fully integrated that includes broiler breeders, broiler houses, layer houses, hatchery, slaughter house, and all necessary facilities for meat and eggs production. Table 2 shows the number of labors and their cost in the eight poultry companies in Kuwait. Revenues of the poultry companies are generated from selling chickens, either live or frozen, and table-eggs. Table 3 shows the sale revues of the poultry farms in Kuwait.

**Table 1:** List of specialized poultry farms that belong to the eight poultry companies in Kuwait.

**Farm no**.	**Broiler (number/yr)**	**Layer (number/yr)**	**Broiler parents (number)**	**Number of houses**	**Farm size**	**Production type**	**Location**
1	8,040,000	373,160	—	67/28	Large	Broiler and layer	Al-Wafra
2	4,494,000	222,480	—	10/43	Large	Broiler and layer	Al-Wafra
3	5,280,000	—	—	41	Large	Broiler production	Amgharah
4	1,200,000	—	—	10	Large	Broiler production	Al-Sulaibiya
5	12,613,200	—	—	54	Large	Broiler production	Al-Wafra
6	111,400	—	—	4	Small	Broiler production	Al-Wafra
7	486,700	—	—	6	Medium	Broiler production	Al-Wafra
8	120,000	—	—	2	Small	Broiler production	Al-Wafra
9	204,000	—	—	6	Medium	Broiler production	Al-Wafra
10	38,130	—	—	3	Small	Broiler production	Al-Wafra
11	—	250,000	—	12	Medium	Layer production	Al-Wafra
12	—	264,800	—	11	Medium	Layer production	Al-Wafra
13	—	99,840	—	4	Small	Layer production	Al-Wafra
14	—	150,000	—	3	Medium	Layer production	Al-Wafra
15	—	68,100	—	2	Small	Layer production	Al-Wafra
16	12,590,032	—	—	18	Large	Broiler production	Al-Abdali
17	—	993,200	—	12	Medium	Layer production	Al-Abdali
18	—	282,880	—	12	Medium	Layer production	Al-Abdali
19	—	—	268,126	25	Medium	Broiler breeders	Al-Abdali
20	18,017,150	372,240	219,860	169	Largest	Broiler breeders/broilers/layers	Al-Shagaiya

*Source: (3, 13) and field visits to the farms (2017)*.

**Table 2:** Number of Labor and their cost in the eight poultry companies in Kuwait.

**Company no**.	**No. of engineers**	**No. of supervisors**	**No. of regular labors**	**Monthly total cost ($)**	**Yearly labor cost ($)**
1.	21	21	184	110,104.620	1,321,255.440
2.	27	27	119	95,574.608	1,146,895.296
3.	8	8	74	43,681.708	524,180.496
4.	2	2	86	37,186.092	446,233.104
5.	1	1	5	3,968.088	47,617.056
6.	2	2	46	21,876.868	262,522.416
7.	1	1	13	6,109.284	73,311.408
8.	1	1	2	2,586.460	31,037.520

**Table 3:** Sale revenues of the poultry farms in Kuwait.

**Company no**.	**Chicken sales ($)**	**Eggs sales ($)**	**Sales revenues ($)**
1.	63,363,686.400	13,750,800.000	77,114,486
2.	55,680,839.424	18,465,360.000	74,146,199
3.	11,722,281.984	1,430,083.200	13,152,365
4.	-	6,836,112.000	6,836,112
5.	2,138,524.416	-	2,138,524
6.	-	5,028,864.000	5,028,864
7.	132,007.680	1,178,640.000	1,310,648
8.	-	392,880.000	392,880

## Results and Discussion

### The Impact pf SARS-Con-V2 on the Local Poultry Companies

As part of this global world, the poultry value chains of eggs and broiler meat of the local companies were also affected by the current COVID 19 crisis. This effect definitely disrupted the supply of poultry products to the consumers in terms of quantity and quality and influenced the production chain at certain points, especially during the partial and full curfew times.

Company no. 1 is the largest poultry company in Kuwait; it owns a series of restaurants and retails that sell poultry products to the public. As the largest local poultry company in Kuwait, the impact of SARS-Con-V2 on company no. 1 started on March 10, 2020 when there was a sudden drop in the consumer demand of poultry products for the restaurants and retails owned by the company. Yet, there is still no infected cases among the workers of the company until writing this paper on June 26, 2020. The main impact of the crisis is caused by the governmental biosecurity curfew measures that disrupted the food distribution chain by restricting movement and distribution of the poultry products. During the full curfew, all the restaurants and selling retails were completely closed. As a result, there has been a sharp decline in the sales of the company and the marketing sector was negatively impacted. As stated by the company manager, the production line was not affected by the SARS-Con-V2 crisis as the company continues to produce its standard production capacity. The stored stock needed for the company to keep production can be enough for 3 months. The company used to export part of its production to the surrounding countries such as Iraq, Kingdom of Saudi Arabia, and other Gulf Countries. The exportation was affected by 10% because of SARS-Con-V2 crisis. Bank closures during the lockdown also affected the availability of the cash money in the company.

Company no. 2 is the second largest poultry company in Kuwait. This company is an integrated company selling table-eggs, live, chilled and frozen chickens and quills, and feed rations to other local poultry companies. This company has layer and broiler poultry farms and poultry processing factory that produces burger, nuggets, frankfurters, meat balls, etc. Although no corona cases were reported among the employees, the impact of COVID 19 crisis on the activities of this company started with the beginning of the full curfew, as all the company retails were completely locked-down. The live broiler chickens and the table-eggs were accumulated in the company farms during the full curfew and this costs the company more feed to keep the broilers alive. These products were marketed in low prices after then to get rid of the accumulated qualitied of the production. The company stock of feed ingredients and vaccination drugs was not affected by the crisis and it is enough for about 3 months. During the full curfew, the loss in sales was 100% for the live birds, 75% for the frozen birds, and 100% for the table-eggs. The partial curfew sales of the table-egg sales, live birds, and frozen birds were reduced by 40, 60, and 40%, respectively.

There was a gradual impact on the sales of company no. 3 starting from March 1, 2020. There was no corona infection among the employees. However, there was movement and transfer restrictions of the employees in between the company farms. The retails and the company were closed during the curfew times. In addition, there was difficulty in purchasing the raw materials and vaccination drugs locally and internationally. Furthermore, the imported goods were piled-up for several days in the port during the full curfew, which could affect the quality of goods and delay the production line. All these factors negatively influenced the production and the sale lines of the company, although the stored material could be barley enough for about 3 months. The company exportation to the surrounding countries was affected by 15% because of the SARS-Con-V2 crisis in the country. The sales in the company retails was affected by 100% during the full curfew and by 40–50% during the partial curfew. Since the company does not have local hatchery eggs for broilers, it was affected by delays in receiving the imported fertilized eggs because of the crisis lockdown.

Company no. 4 mainly produces table-eggs to the society. The negative impact of the corona virus on company no. 4 started on March 1, 2020. The production line was not influenced, but the main impact was on the marketing and sales line. The stored raw material for production could be enough for about 3 months from June 1, 2020. The company manager stated that no corona-positive employees were noticed. The governmental partial and full curfew negatively influenced the marketing process of the table-eggs, as the consumer demand sharply dropped. Closure of the company retails during the curfew and the difficulties of employee transportation were the main difficulties facing the company. There was 100% loss in the table-eggs produced during the full curfew, as the production could not be sold to the bakery shops and the public. The main concern of the company management is the rise in the price of the imported raw materials and vaccinations needed for the laying hens. In addition, half of the company's production of table-eggs was exported to the surrounding countries such as Iraq and the Gulf Countries before the SARS-Con-V2 crisis. After the crisis, there is exportation ban and border closure that affect the transportation process. The company had to lower the table-eggs price and lower the production capacity.

Company no. 5 is specialized in broiler chicken production only. There was a serious impact of SARS-Con-V2 crisis on the activity of company no. 5 starting from March 1, 2020. Although there was no infectious among workers of the company, but the company obliged to completely terminate all its production series and activities. This cease was because the company does not have enough stock of raw materials and feed ingredients to keep production as the government restricted the importation process. As a result, there was 100% loss of sales and profit.

Company no. 6 is specialized in egg and fodder production, from which 70% was sold in the local markets, while about 30% was exported to the gulf countries and Iraq. This company was also influenced by the current COVID 19 crisis. This influence was started early of March in the form of delays in imported cargo for the raw material for feed ration production, as well as sharp increases in the transportation and importation prices. As a result, the company stock was critically influenced and it will be barley enough for only 1 month. The company exports were totally terminated due to the lockdown and ban of all the transportation and exportation activities between the surrounding countries. The loss in egg sales was 40% during the full curfew and 30% during the partial curfew.

The impact of COVID 19 on company 7 started with the full curfew on May 10, 2020. As with the other poultry companies, the main impact was on the sales sector during the full curfew because of the full lockdown and movement and transportation restrictions. This resulted in increase in the broiler chicken weights at slaughter, which is not preferable by the consumers in Kuwait. This obliged the company to reduce the sale prices and thus loss in profits. Fortunately, there was no impact of the crisis on company sales during the partial curfew. This company was used to sell feed ingredients to other local poultry companies, but because of the stock shortage, the company stopped providing other companies with feed ingredients supply and limited the supply to its own production. The company has no self-retails to sell its products and it does not export its products to other countries. It sells the live broiler chickens and table-eggs to the local outlets that were completely closed during the full curfew, which considerably affected the sales and profit.

The impact of COVID 19 virus on company 8 started in early April, in the form of delayed cargo of the raw feed ration ingredients from other countries. As with other local poultry companies, the activities of the company was also impacted by the partial and full curfews that resulted in to restrictions in transportation of products and movement of staff. Consequently, this influenced the sales and the production lines of the company. The company was obliged to buy the feed ingredients locally from other poultry company, as there was no stock of raw materials. Changing the source of the raw feed ingredients affected the quality of the feed rations, and definitely affected the quality of egg production. This farm is specialized in table-egg sales that was reduced by 65% during the full curfew and by 50% during the partial curfew. Home delivery was totally stopped during the lockdown (100%) and the sales were only in the company retail shopping center.

**FIGURE 1:**
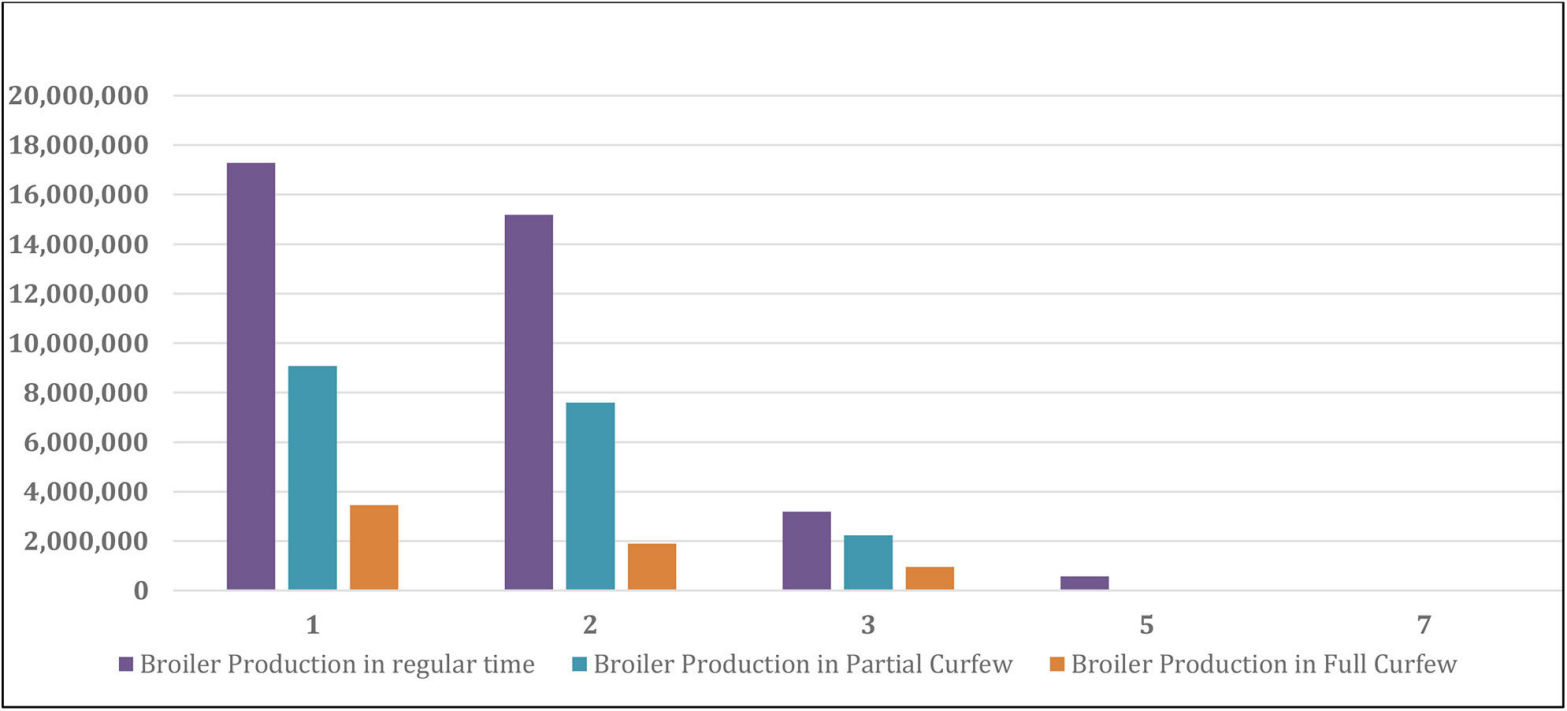
Broiler production (bird/year) before and after SARS-Con-V2 crisis.

**FIGURE 2:**
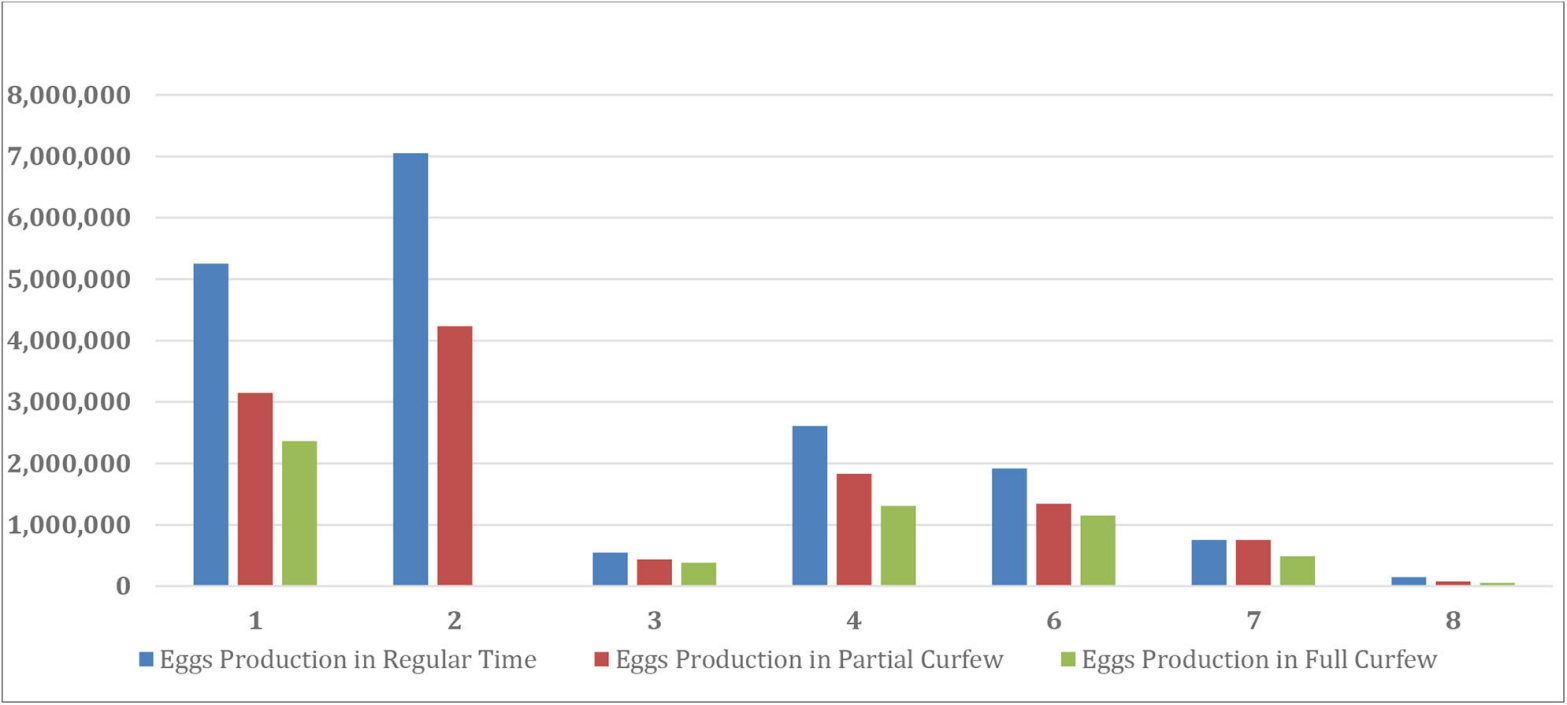
Eggs production (tray/year) before and after SARS-Con-V2 crisis.

**FIGURE 3:**
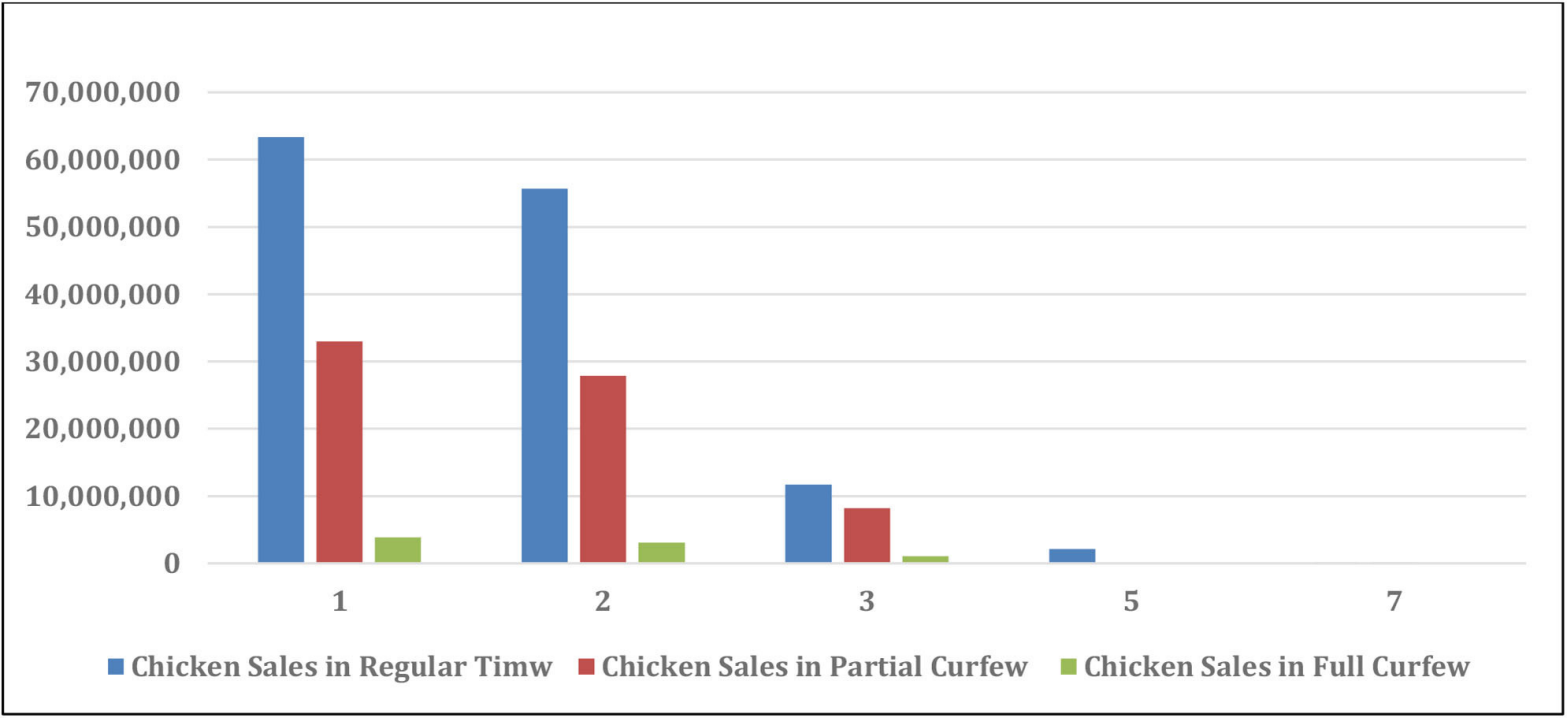
Chicken sales in USD before and after SARS-Con-V2 crisis.

**FIGURE 4:**
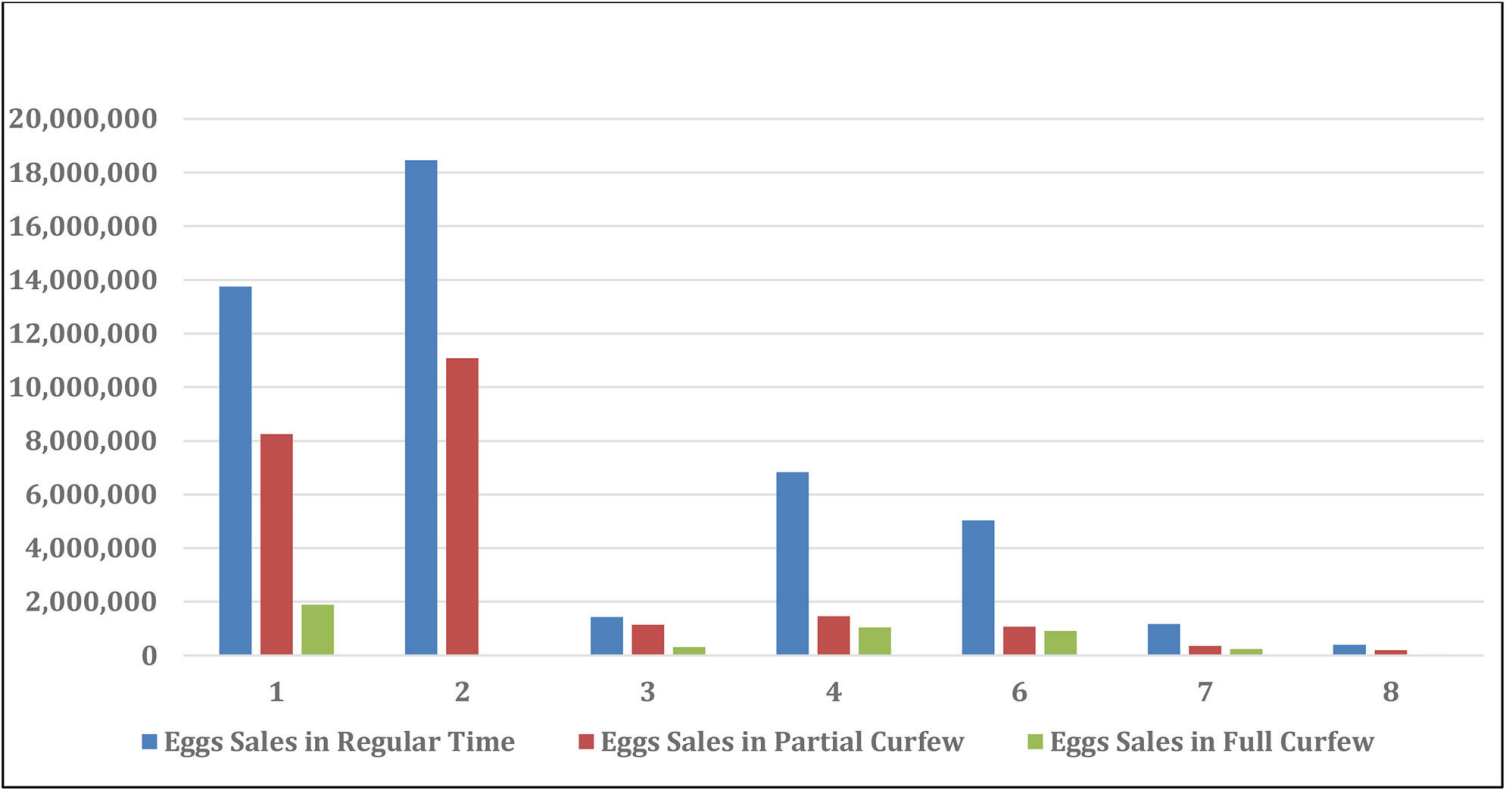
Egg sales in USD before and after SARS-Con-V2 crisis.

Generally speaking, the SARS-Con-V2 crisis impacted all the local poultry companies by restricting movements and transportation of the employees in between the company branches, exportation and importation of goods, bank closures, rise in the imported and local vaccination and drug prices, sales and marketing. The effect of the crisis partial and full curfews on the sales loss of live and frozen broiler chickens and table-eggs is shown in Table 4. The sales size after SARS-Con-V2 crisis in the eight poultry farms in Kuwait is shown in Table 5. The effect of SARS-Con-V2 crisis on the sales loss during the partial and full curfews is shown in Table 6. Table 7 summarizes the average percentage loss in broiler chicken and table-egg sales during the partial and full curfews. The results of Table 7 show that the largest percent loss was in the broiler chicken sales during both the partial and full curfews, and that more loss was observed during the full curfew than the partial curfew. To further elucidate this effect, graphical illustrations are shown in Figures 1–4 to display the effect of the SARS-Con-V2 crisis partial and full curfews on the poultry industry in Kuwait. Figure 1 shows the broiler Size of Production before and after the crisis. The table-eggs production before and after the crisis is shown in Figure 2. Figures 3, 4 show the effect of the crisis on the broiler chicken and table-egg sales during partial and full curfew, respectively.

**Table 4:** The effect of the crisis partial and full curfews on the sales loss of live and frozen broiler chickens and table-eggs.

**Company No**.	**Company size**	**% loss in sales**					
		**Partial curfew time**			**Full curfew time**		
		**Live chickens**	**Frozen chickens**	**Table-eggs**	**Live chickens**	**Frozen chickens**	**Table-eggs**
1.	Large	50	45	40	100	60	55
2.	Large	60	40	40	100	75	100
3.	Large	40	20	20	100	40	30
4.	Medium	NA	NA	30	NA	NA	50
5.	Medium	100	100	NA	100	100	NA
6.	Medium	NA	NA	30	NA	NA	40
7.	Small	0	0	0	85	85	35
8.	Small	NA	NA	50	NA	NA	65

**Table 5:** Sale size after the crisis in the eight poultry farms in Kuwait.

**Company no**.	**Sales in partial curfew time**			**Sales in full curfew time**		
	**Chicken sales ($)**	**Eggs sales ($)**	**Sales revenues ($)**	**Chicken sales ($)**	**Eggs sales ($)**	**Sales revenues ($)**
1.	32,949,116.928	8,250,480	41,199,596.928	12,672,737.280	6,187,860.000	18,860,597.28
2.	27,840,419.712	11,079,216	38,919,635.712	10,022,552.144	-	10,022,552.144
3.	8,205,596.734	1,144,067	9,349,663.294	3,516,685.250	1,001,058.240	4,517,743.490
4.	-	4,785,278	4,785,278.400	-	3,418,056.000	3,418,056.000
5.	-	-	-	-	-	-
6.	-	3,520,205	3,520,204.800	-	3,017,318.400	3,017,318.400
7.	132,007.680	1,178,640	1,310,647.680	19,801.152	766,116.000	785,917.152
8.	-	196,440	196,440.000	-	137,508.000	137,508.000

**Table 6:** Sales loss as an effect of SARS-Con-V2 in the poultry companies in Kuwait.

**Company no**.	**Sales loss in partial curfew ($)**	**Sales loss in full curfew ($)**
1.	35,914,889.472	58,253,889.120
2.	35,226,563.712	64,123,647.280
3.	3,802,701.890	8,634,621.694
4.	2,050,833.600	3,418,056.000
5.	2,138,524.416	-
6.	1,508,659.200	2,011,545.600
7.	-	524,730.528
8.	196,440.000	255,372.000

**Table 7:** Average percentage loss in chicken and table-egg sales during partial and full curfews.

**Average % of loss in partial curfew time**		**Average % of loss in full curfew time**	
**(Live + frozen chickens)**	**Eggs**	**(Live +frozen chickens)**	**Eggs**
57%	30%	85%	54%

### Key Challenges to the Local Poultry Industry During the COVID 19 Crisis

Based on the above results of the survey among the poultry companies in Kuwait, it is obvious that the main challenge of the global quarantine and curfews because of COVID 19 crisis is the difficulty of transportation of feed ingredients among countries. This severely influenced the availability of the main ingredients needed for the feed rations, especially that most of the feed ingredients such as corn and soybean are not produced locally and have to be imported from other countries (14). In addition, some areas were totally quarantined by the Kuwait Government such as Hawally, Khetan, Farwaneyya, Mahbola, and Jeleeb Shuyookh. This resulted into total restriction of the workers movement for long period of time, which considerably affected the smoothness of workflow in the company farms.

Furthermore, the global and local quarantine considerably impacted the quality and price of the raw feed ingredients, whether imported or locally purchased from other poultry companies. The shortage in availability of raw material increases the cost, regardless of the quality standards. Changing the type and quality of feed ingredients would affect the production performance of chickens if they are used to certain quality of feed rations. All of these challenges play an important role in production cycle and considerably influence the food supply chain of table-eggs and meat to the consumer.

### Proposed Solutions to Combat COVID 19 Challenge

First of all, and in order to further identify the impact of COVID 19 on the local poultry industry, a poultry value chain of the local poultry industry should be first developed and then analyzed to make it easy for identification of inefficiencies in the value chain and the areas where the industry could be improved. Accordingly, the impact of COVID 19 crisis could be identified and mitigated through better processes or introduction of new technologies. This local poultry value chain will make it easy to transfer technology to the private sector to fill gaps in the food value chain. In addition, it will provide strong combat against the current corona virus or any future global or national crisis. As a result, a sustainable production of poultry products and a tangible contribution to the national food security will be achieved.

Application of strict biosecurity measures including full and partial curfew is one of the effective approaches to combat and reduces incidences of COVID 19. Although there are no reported cases of the corona virus transfer to chickens, optimizing the immune status of the birds in the farm is also a major priority to keep the health of the flocks at optimum to face any other outbreaks and infectious agents. This includes execution of a strict health program to avoid occurrence of viruses such as avian influenza or any other respiratory avian diseases (15, 16). The elements of a good health program are good vaccination, disinfecting, cleaning, continuous monitoring, and pest control (1).

As a solution to the importation ban and global feed ingredients shortage, the possibility of the local production of the main feed ingredients such as corn and soya should be explored. In addition, any other local sources of protein and energy could be used to partially replace corn and soya in the feed rations. Examples of the local sources are marine algae, whole dates, date pits, and native plants in the country. This approach will definitely contribute to provide local feed ingredients in the absence of imported sources due to global crisis such as the current COVID 19 crisis (14, 17, 18).

Furthermore, the production efficiency should be improved by applying the up-to date technologies and know-how to improve the production performance parameters and to compete with the imported products in terms of quality and price. A technology transfer model should be identified between the poultry research institutes such as Kuwait Institute for Scientific Research (KISR) and the poultry stakeholders in the country. Such model will provide a chance for KISR to recognize the main challenges opposing the sector and try to find solutions to these challenges. Therefore, a Memo of Understanding (MOU) was developed between KISR and the largest poultry company in Kuwait to prepare poultry development plan in collaboration with the company to ensure the benefit of the sector from KISR's R&D results.

It is essential to notice that only two local poultry companies produce their own hatching eggs for 1-day old chicks. Most of the poultry companies depend totally on external sources to import their needs of the hatching eggs and the 1-day-old chicks. As a result, other strategies should be implemented to mitigate the dependence on the imported hatching egg and 1-day-old pullets for broiler meat and table eggs production. One of these strategies is the development of a central breeding company that applies high quality breeding production programs to produce the hatching eggs and to provide all the local companies with these products to minimize the importation process. Additionally, developing a breeding program to produce a pure local chicken breed in Kuwait via selection and breeding programs is another solution to a void complete dependence on the imported hatching eggs.

In addition to the local production of broiler meat, table-eggs and hatching eggs, diversifying the local poultry products such as local chickens, quails, ostriches, and ducks will provide a solution to enhance the poultry products in Kuwait and to contribute to food security in the country. Establishing a food security network between the Gulf Countries (GCC) will be a good solution to combat trade restrictions with other far away countries. This network will ensure sustainable flow of products between the GCC countries during global crisis.

## Conclusion and General Recommendations

Poultry industry in Kuwait was considerably affected by the current global COVID-19 crisis. This effect was mainly because of global and local transportation bans, as well as lockdown during the partial and full curfews. The following recommendations maybe applied to mitigate the effect of the current and future crisis on the poultry industry:

- Establishment of a poultry value chain in Kuwait.
- Vertical improvement through R & D and technology transfer by local research institutions.
- Minimize dependence on imported products by local utilization of resources.
- Enhancing diversity of poultry products.
- Encourage the integrated relationships between research institutes, policy makers, stakeholders and other bodies related to food security.
- Horizontal investment by expanding land space for integrated poultry farming.
- Establishment of food security network between the GCC countries.

## Data Availability Statement

The original contributions presented in the study are included in the article/supplementary material, further inquiries can be directed to the corresponding author/s.

## Author Contributions

HA-K is the main author of the manuscript. The other authors assisted in data collection and preparation of the figures. AA-N supervised and reviewed the work. All authors contributed to the article and approved the submitted version.

## Conflict of Interest

The authors declare that the research was conducted in the absence of any commercial or financial relationships that could be construed as a potential conflict of interest. The handling Editor declared a past co-authorship with one of the authors HA-K.

## References

[1] Sireesha P Prasanna S Designer eggs and poultry meat as functional foods–an overview. Pharm Innov J. (2019) 8:829–31.

[2] Al-Khalaifa H Al-Nasser A Al-Surayee T Al-Kandari S Al-Enzi N Al-Sharrah T. Effect of dietary probiotics and prebiotics on the performance of broiler chickens. Poult Sci. (2019) 98:4465–79. 10.3382/ps/pez282 31180128

[3] Al-Nasser A Al-Khalaifah H Al-Mansour H Ahmad A Ragheb R Evaluating farm size and technology use in poultry production in Kuwait. World Poultry Sci J. (2020) 1–16. 10.1080/00439339.2020.1737625

[4] FAO Declaration on World Food Security. Rome: World Food Summit, FAO (1996).

[5] McLeod A Kobayashi M Gilman J Siagian A Young M The use of poultry value chain mapping in developing HPAI control programmes. World Poultry Sci J. (2009) 65:217–24. 10.1017/S0043933909000166

[6] Weersink A von Massow M McDougall B Economic thoughts on the potential implications of COVID-19 on the Canadian dairy and poultry sectors. Can J Agr Econ. (2020) 68:195–200. 10.1111/cjag.12240

[7] Al-Khalaifah H Al-Nasser A Givens D Rymer C Yaqoob P. Comparison of different dietary sources of n-3 polyunsaturated fatty acids on immune response in broiler chickens. Heliyon. (2020) 6:e03326. 10.1016/j.heliyon.2020.e03326 32051880 PMC7002886

[8] Al-Nasser A Al-Khalaifa H Al-Bahouh M Khalil F Ragheb G Challenges facing poultry production in Kuwait. World Poultry Sci J. (2015) 71:339–48. 10.1017/s0043933915000343

[9] Taha M Henney MT Kuwait Poultry and Products Annual Poultry Meat Report. Foreign Agriculture Service/USDA. Global Agriculture Information Network. Gain report No. KU5003 (2005). p. 1–9.

[10] Al-Khalaifah HS. Benefits of probiotics and/or prebiotics for antibiotic-reduced poultry. Poult Sci. (2018) 97:3807–15. 10.3382/ps/pey160 30165527

[11] USDA FAS Livestock and Poultry: World Markets and Trade. April, 2014. Foreign Agricultural Service/USDA. Office of Global Analysis (2014). Available online at: www.fas.usda.gov/data/livestock-and-poultry-world-markets-and-trade (accessed November 07, 2018).

[12] Wikipedia contributors COVID-19 Pandemic in Kuwait: Wikipedia, The Free Encyclopedia. (2020) Available online at: https://en.wikipedia.org/w/index.php?title=COVID-19_pandemic_in_Kuwait&oldid=960407263 (accessed March 01, 2020).

[13] Public Authority for Agriculture and Fish Resources Poultry Section, Chicken Disease Section for the Period January 1, 2016 to December 31, 2016. Kuwait: The Public Authority for Agriculture Affairs and Fish Resources (2016).

[14] Al-Khalifa H Al-Nasser A Al-Bahouh M Ragheb G Al-Qalaf S Al-Omani N The effect of polyunsaturated fatty acids on avian immune cell subpopulations in peripheral blood, spleen, and thymus. World Poultry Sci J. (2016) 72:531–4. 10.1017/s0043933916000428

[15] Abdel-Hafez M Mohamed MJZVJ Evaluation of some immunostimulants on the immune-response of broiler chickens against avian influenza and newcastle diseases vaccination. Zagazig Vet J. (2016) 44:273–81. 10.21608/zvjz.2016.7881

[16] Hafez HM Attia YA Challenges to the poultry industry: current perspectives and strategic future after the COVID-19 outbreak. Front Vet Sci. (2020) 7:516 10.3389/fvets.2020.00516 33005639 PMC7479178

[17] Al-Khalifa H Enrichment of poultry diets with polyunsaturated fatty acids (PUFA) for human consumption. APDV. (2017) 1 10.31031/APDV.2017.01.000523

[18] Al-Nasser A Al-Khalaifah H Khalil F Al-Mansour H Poultry industry in the Gulf Cooperation Council with emphasis on Kuwait. World Poultry Sci J. (2020) 1–13. 10.1080/00439339.2020.1782802

